# Machine Learning Algorithms for the Diagnosis of Class III Malocclusions in Children

**DOI:** 10.3390/children11070762

**Published:** 2024-06-24

**Authors:** Ling Zhao, Xiaozhi Chen, Juneng Huang, Shuixue Mo, Min Gu, Na Kang, Shaohua Song, Xuejun Zhang, Bohui Liang, Min Tang

**Affiliations:** 1 Department of Orthodontics, Guangxi Medical University College of Stomatology, Nanning 530021, China; kq100611@sr.gxmu.edu.cn (L.Z.); nanlan@sr.gxmu.edu.cn (S.M.); 430046@sr.gxmu.edu.cn (N.K.); 430036@srgxmu.edu.cn (S.S.); 2 Department of Stomatology, Guangxi Chinese-Traditional Medical University, Nanning 530021, China; chenxz@gxtcmu.edu.cn; 3 School of Computer, Electronics and Information, Guangxi University, Nanning 530004, China; 2213391004@st.gxu.edu.cn (J.H.); xjzhang@gxu.edu.cn (X.Z.); liangbh@st.gxu.edu.cn (B.L.); 4 Department of Paediatric Dentistry and Orthodontics, Faculty of Dentistry, University of Hong Kong, Hong Kong SAR, China; drgumin@hku.hk; 5 Guangxi Clinical Research Center for Craniofacial Deformity, Nanning 530021, China

**Keywords:** children, Class III malocclusion, machine learning, diagnosis, feature importance analysis

## Abstract

Artificial intelligence has been applied to medical diagnosis and decision-making but it has not been used for classification of Class III malocclusions in children. Objective: This study aims to propose an innovative machine learning (ML)-based diagnostic model for automatically classifies dental, skeletal and functional Class III malocclusions. Methods: The collected data related to 46 cephalometric feature measurements from 4–14-year-old children (*n* = 666). The data set was divided into a training set and a test set in a 7:3 ratio. Initially, we employed the Recursive Feature Elimination (RFE) algorithm to filter the 46 input parameters, selecting 14 significant features. Subsequently, we constructed 10 ML models and trained these models using the 14 significant features from the training set through ten-fold cross-validation, and evaluated the models’ average accuracy in test set. Finally, we conducted an interpretability analysis of the optimal model using the ML model interpretability tool SHapley Additive exPlanations (SHAP). Results: The top five models ranked by their area under the curve (AUC) values were: GPR (0.879), RBF SVM (0.876), QDA (0.876), Linear SVM (0.875) and L2 logistic (0.869). The DeLong test showed no statistical difference between GPR and the other models (*p* > 0.05). Therefore GPR was selected as the optimal model. The SHAP feature importance plot revealed that he top five features were SN-GoMe (the ratio of the length of the anterior skull base SN to that of the mandibular base GoMe), U1-NA (maxillary incisor angulation to NA plane), Overjet (the distance between two lines perpendicular to the functional occlusal plane from U1 and L), ANB (the difference between angles SNA and SNB), and AB-NPo (the angle between the AB and N-Pog line). Conclusions: Our findings suggest that ML models based on cephalometric data could effectively assist dentists to classify dental, functional and skeletal Class III malocclusions in children. In addition, features such as SN_GoMe, U1_NA and Overjet can as important indicators for predicting the severity of Class III malocclusions.

## 1. Background

A Class III malocclusion, as classified by Edward H. Angle, is a type of malocclusion characterized by anterior teeth in crossbite or reverse overjet, meaning the upper front teeth are positioned behind the lower front teeth, often with the molars engaged in a mesial relationship [1]. Based on the etiology, severity, and prognosis, it can be categorized into three types: dental, skeletal, and functional. The dental Class III tpye refers to an anterior crossbite caused solely by abnormal positioning of the upper and lower incisors. The functional Class III type is an acquired condition involving neuromuscular participation, resulting in the forward positioning of the mandible. The skeletal Class III type is due to imbalanced growth between the upper and lower jaws, often presenting as excessive mandibular development, which is difficult to correct and may require surgery in severe cases [1]. The global prevalence of Class III malocclusions exceeds 7%, with even higher rates in Southeast Asia, ranging from 12.58 to 26.67% [2]. Class III malocclusions can significantly affect oral functions, facial aesthetics and psychological health in children. Early symptoms of anterior crossbite are not very noticeable, but as children age, the deformities can worsen, making correction increasingly difficult [3,4]. Therefore, early diagnosis and treatment are crucial [5,6,7].

The severity and prognosis of different types of Class III malocclusions necessitate varied early intervention strategies. However, due to the complex and diverse underlying alveolar structures, functional crossbites often coexist with varying degrees of skeletal anomalies, and skeletal crossbites may also present functional factors. Since these two factors often coexist, a strict clinical differential diagnosis between functional and skeletal crossbites is not easy, making the classification and differential diagnosis of Class III malocclusions in children a challenging orthodontic issue.

Previous research has primarily focused on developing various analytical models to summarize the radiographic features specific to Class III malocclusions, and thus aiding their diagnosis. Schulhof et al., (1977) [8] established a simple formula based on lateral cephalometric measurements to predict the facial growth in skeletons of patients with Class III malocclusions. Subsequently, scholars attempted to identify meaningful indicators for the diagnosis and treatment of Class III malocclusions using conventional statistical models such as cluster analysis, discriminant analysis and regression analysis [9,10,11]. However, despite extensive research on diagnostic indicators, the findings were inconsistent and satisfactory accuracy metrics remained a challenge [12].

In recent years, artificial intelligence (AI) has found widespread applications in healthcare and dentistry where they have enhanced diagnostic accuracy and clinical decision-making [13,14,15,16,17,18,19,20,21]. Machine learning (ML) is the core method for implementing AI, and it involves algorithmic analysis of datasets to learn and make corresponding decisions and predictions for real-world events [22]. Previous studies have demonstrated that different ML methods can effectively assist orthodontists in diagnosis as well as to standardize diagnostic criteria and improving healthcare efficiency. These methods include identifying cephalometric landmarks [23,24,25,26,27,28,29,30,31] and making decisions regarding tooth extractions [32,33,34,35,36,37]. Previously, we developed an AI model capable of automatically classifying sagittal facial bone patterns in children with malocclusions, thereby achieving a diagnostic accuracy of up to 93% on the validation set [38]. Additionally, ML has been applied to diagnose and treat adult Class III malocclusions, resulting in enhanced predictive accuracy [39]. Kim et al., (2009) compared ML algorithms with traditional discriminant analysis for predicting treatment outcomes in pediatric Class III malocclusions, and suggested that this technique could effectively replace traditional prognostic models [12]. However, research specifically related to ML-based classification diagnosis of Class III malocclusions in children remain scarce.

Consequently, this study has compiled a comprehensive dataset of pediatric Class III malocclusion cases. Utilizing this dataset, we trained ten machine learning models: K-Nearest Neighbor (KNN), Logistic Regression (LR), Linear Support Vector Machine (Linear SVM), Radial Basis Function Support Vector Machine (RBF SVM), Gaussian Process, Decision Tree (DT), Multilayer Perceptron (MLP), Random Forest (RF), Quadratic Discriminant Analysis (QDA) and Extreme Gradient Boosting (XGBoost). We validated the predictive performance of these models and compared their accuracies to identify the most suitable model for predicting the type of Class III malocclusion. Furthermore, we conducted an in-depth analysis of the training network to determine the most critical phenotypic features affecting diagnostic classification. This will provide clinicians with more objective diagnostic tools that would be capable of accurately diagnosing early developmental Class III malocclusions in children and assist in timely and appropriate treatment regimens.

## 2. Materials and Methods

**Figure 1:**
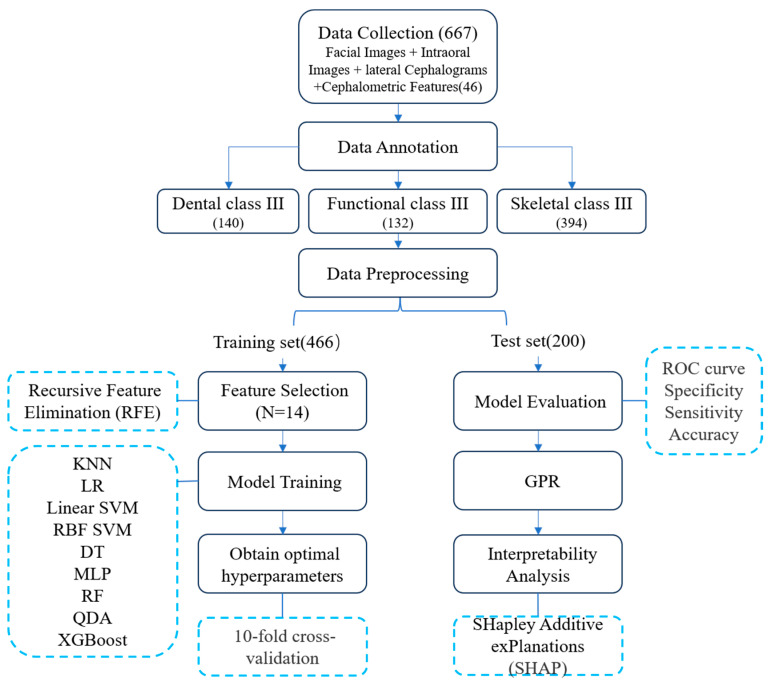
General workflow diagram for the machine learning protocol used for the classification and diagnosis of patients with Class III malocclusions.

This study was exempted from IRB approval and this was confirmed by the Ethics Committee of Guangxi Medical University in 26 July 2023 (Approval No. 2023-KY0167). All procedures were conducted in accordance with applicable regulations and the methodology used in this study is presented in Figure 1.

### 2.1. Data Collection

This retrospective cross-sectional study investigated orthodontic cases by using detailed searches of previously gathered electronic dental records. We collected clinical data from pediatric malocclusion patients who met the inclusion criteria. All the patients had attended the School of Stomatology, Guangxi Medical University, from January 2002 to December 2022. The dataset included facial images as well as medical records. The total number of images in the dataset was approximately 6660, and these comprised of facial and intraoral photographs, and lateral cephalometric radiographs. We captured lateral cephalometric radiographs with a Myriad Hyperion X9 (Safelite Group, Cormano, Italy), with original images of 2460 × 1950 or 1752 × 2108 pixels at 0.1 mm/pixel resolution. In addition, we used a Nikon D7200Nikon Corporation, Tokyo, Japan ) to capture photographs of patients with an original resolution of 2000 pixels and a resolution of 0.1 mm. All images obtained were in the JPG format.

Inclusion Criteria:

- Age range: Children aged from the age of 4 to 14 years old
- Dental arch condition: When cusps were interdigitated, anterior teeth present with crossbite or negative overjet, and molar relationships were either Class I or Class III.
- Craniofacial symmetry: Good, with hard tissue pogonion deviation from the midline ≤1 mm
- Complete clinical and imaging data, with all images clear and intact

Exclusion Criteria:

- Severe systemic diseases
- Craniofacial syndromes, craniofacial defects or significant asymmetrical deformities
- History of orthodontic treatment, trauma or surgery
- Impacted maxillary central incisors

### 2.2. Cephalometric Measurement Analysis

We performed cephalometric measurements on pre-treatment lateral cephalometric radiographs using iortho 10.1 software. Prior to any measurements being taken, an experienced orthodontist from the School of Stomatology, Guangxi Medical University, trained three orthodontists, and periodic calibration was conducted on 8% of the sample. The dataset included 46 cephalometric measurement features which are defined in Table 1. The cephalometric landmarks are detailed in Figure S1.

### 2.3. Data Annotation

After reviewing medical history, assessing clinical data, and analyzing cephalometric measurements, we annotated the classification diagnosis of Class III malocclusion. All diagnoses were carefully determined by three experienced orthodontists with 20 years of clinical experience (Table 2). In cases where two experts had differing judgments for the same patient, the case was discussed among all experts to reach a consensus.

### 2.4. Data Preprocessing and Feature Selection

Firstly, we pre-processed all data referring to features within the dataset to ensure that each feature had a mean of 0 and a standard deviation of 1. Next, we randomly divided all the study subjects into training (*n* = 466) and testing (*n* = 200) sets at a 7:3 ratio. In the training set, we employed the Recursive Feature Elimination (RFE) algorithm in order to perform feature selection on the 46 input parameters.

### 2.5. Model Training and Evaluation

We constructed ten ML learning models, including K-Nearest Neighbor (KNN), logistic regression (LR), linear support vector machine (Linear SVM), radial basis function support vector machine (RBF SVM), Gaussian process, decision tree (DT), multilayer perceptron (MLP), random forest (RF), quadratic discriminant analysis algorithm (QDA) and extreme gradient boosting (XGBoost). Using the filtered feature data, we trained each of these models separately and performed grid searches with ten-fold cross-validation to obtain the optimal hyper-parameters. Finally, we independently validated the models on the testing set and determined the best model by comparing the area under the receiver operating characteristic (ROC) curves using the DeLong test.

### 2.6. Interpretability Analysis

We performed interpretability analysis on the best model by using the SHapley Additive exPlanation (SHAP) tool for understanding the importance and impact of input features on output decisions.

## 3. Results

### 3.1. Baseline Data Analysis

We summarized the clinical histories of all study subjects from January 2002 to December 2022. A total of 666 pediatric patients were included in this study, with an age range of 4–14 years (mean age = 10.68 ± 2.04 years). Among them, there were 357 males and 309 females. The average ages differed significantly among the three groups (*p* < 0.05), with those in the skeletal Class III malocclusion group having the highest mean age. According to expert diagnoses, the proportions of dental, functional and skeletal Class III malocclusions were 21.02, 19.82 and 59.16%, respectively, with last parameter being predominant (Table S1).

Pairwise comparisons revealed statistically significant differences (*p* < 0.05) in most cephalometric measurement indices between the skeletal group and both the dental and functional groups. In the comparison between the dental and functional groups, statistically significant differences (*p* < 0.05) were mainly observed in indices representing dental and alveolar features. Non-significant differences (*p* > 0.05) were more common in indices related to craniofacial and soft tissue features. Among the dental, skeletal and functional groups, several indices, including age, ANB, NA-APo, SN-GoMe, Go-Pog, SGn-FH, AB-NPo, Wits, Overjet, U1-NA, U1-Apo (mm), L1-Apo (mm), IMPA and Si-H, were significantly different (*p* < 0.05). However, except for L1-Apo (mm) and Si-H, there were no differences between the dental and functional groups (*p* > 0.05). Notably, indices such as ANB, AB-NPo, NA-APo, Go-Pog, Wits, Overjet and L1-Apo (mm), which reflected the severity of Class III malocclusions, had higher average values in the skeletal group of patients (Table S2).

### 3.2. Model Establishment and Performance Evaluation

**Figure 2:**
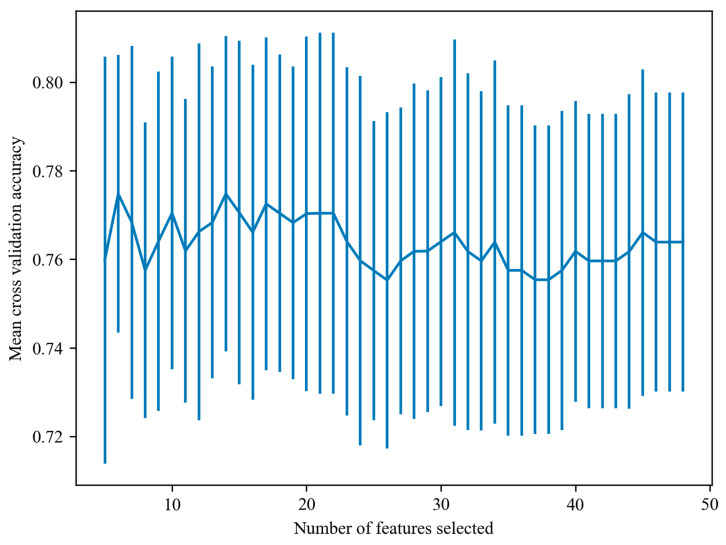
A recursive feature elimination curve.

We employed the RFE algorithm to eliminate redundant features, reduce feature dimensions and select the optimal feature combination. Initially, we constructed ML models by training them on all 46-dimensional features and evaluated their average accuracy. Subsequently, based on feature importance assessment, we iteratively removed the least important features. When the feature count was 14, the model achieved the highest average accuracy on the validation set (Figure 2). The specific selected features are detailed in Table S3.

**Figure 3:**
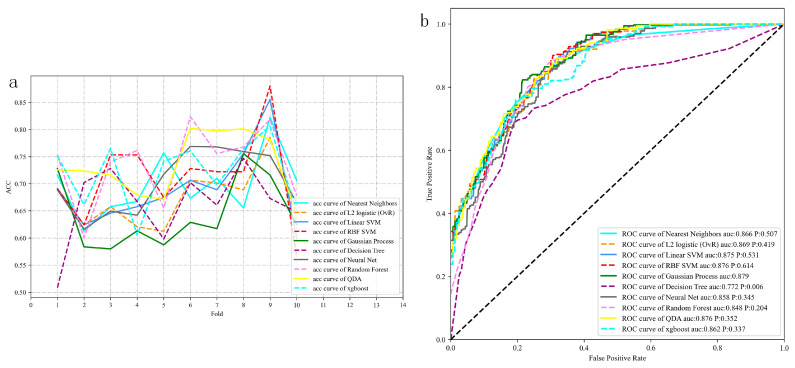
The acc (**a**) and ROC curves (**b**) for the 10 models.

By utilizing ten-fold cross-validations, we fine-tuned the hyper-parameters for ten ML models, and the optimal parameter combinations were determined for each model (Figure 3a). We then applied the trained models to the testing set, and the ROC curves obtained for the ten models are shown in Figure 3b. The top five models ranked using their area under the curve (AUC) values were Gaussian process regression (GPR; 0.879), radial basis function support vector machine (RBF SVM; 0.876), quadratic discriminant analysis (QDA; 0.876), linear SVM (0.875) and L2 logistic (OvR; 0.869). The DeLong test showed no significant differences (*p* > 0.05) between GPR and the other models, including KNN, OvR, linear SVM, RBF SVM, Neural Net, QDA and XGBoost. The overall performance of the ten ML prediction models is summarized in Table 3.

Therefore, based on the AUC value, we selected GPR as the optimal model. GPR is a supervised ML technique suitable for classification and regression tasks. It utilizes the Gaussian process prior to regression of the analyzed data and determines the kernel function parameters by using Bayesian posterior probabilities and maximum likelihood estimations.

### 3.3. Interpretability Analysis

**Figure 4:**
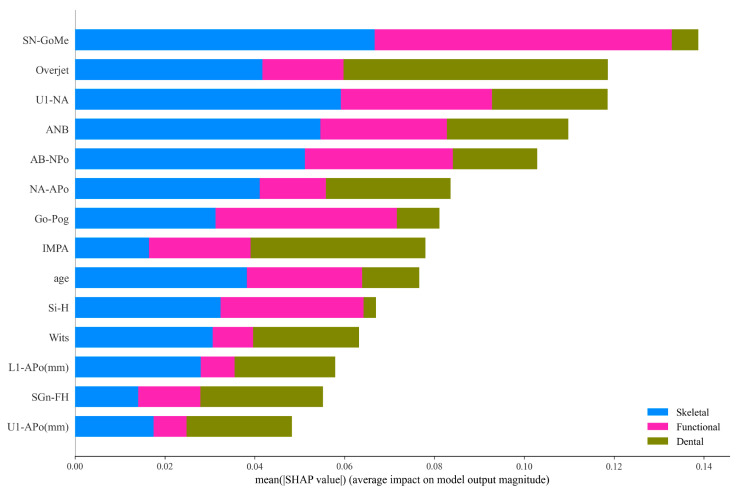
Feature importance for the Class III classification. A total of 14 features are shown with respect to age and 12 cephalometric items were used for classification.

**Figure 5:**
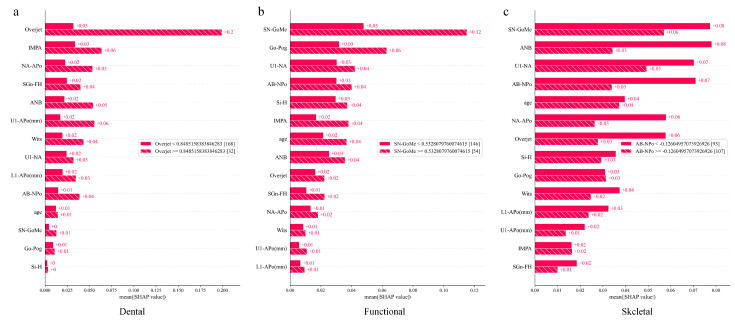
The feature importance for the different classification categories. (**a**) The feature importance for dental Class III malocclusion. (**b**) The feature importance for functional Class III malocclusion. (**c**) The feature importance for skeletal Class III malocclusion.

We conducted an interpretability analysis on the GPR, which was deemed to be the best model, by using the SHAP tool. The feature importance plot (Figure 4) illustrates the average importance ranking of input features for predicting the classification outcomes. The top five features were SN_GoMe, U1_NA, Overjet, ANB and AB_NPo. However, feature importance rankings differed across the different classification categories. Figure 5a–c are three horizontal bar charts that illustrate the impact of different features on the Gaussian Process model. Each bar chart’s vertical axis represents a feature, while the horizontal axis indicates the average absolute value of that SHAP feature. Figure 5a shows the influence of different features on the Gaussian Process classification model when Dental was considered the positive class (the category of interest) and the rest as the negative classes. Such analysis helps reveal which features play key roles in distinguishing the first category from the others. Here, the Overjet feature was identified as a key feature due to its significant difference in distinguishing Dental Class III malocclusions. Figure 5b and Figure 5c, respectively, represent the feature bar charts when Functional and Skeletal were considered as the positive classes. We can see that the top three important features for distinguishing whether it is Functional are: SN_GoMe, Go_Pog and U1_NA, while for distinguishing whether it is Skeletal, the top three important features are: SN-GoMe, ANB and U1_NA (Figure 5).

## 4. Discussion

The classification diagnosis and early treatment of pediatric Class III malocclusions pose complex challenges in orthodontics. Class III malocclusions have a tendency to worsen, as children grow and develop, leading to increased difficulties during treatment. Proper and timely early interventions are crucial for minimizing later treatment challenges. Experienced dentists will always advise for early diagnosis and treatment of Class III malocclusions. However, current classification relies primarily on subjective judgments by orthodontists, and it lacks universally accepted features and indicators of this condition. Therefore, a new, more accurate method for classifying pediatric Class III malocclusions is needed. Although ML applications in orthodontics are gaining attention, there has been no specific studies that apply ML to pediatric Class. III malocclusion classification. In this study, we propose an innovative ML-based model for classifying pediatric Class III malocclusions by using previously obtained pre-treatment cephalometric measurements. The model automatically categorizes malocclusions into dental, skeletal and functional classes, and it also analyzed the importance of included evaluation indices to assist in future clinical diagnoses.

Automated diagnostic tools based on AI are gaining widespread attention as practical clinical aids and represent a growing trend in orthodontics. ML has been used for diagnosing and treating Class III malocclusions. Fudalej et al. [40] reviewed the important predictive factors for early orthodontic and orthopedic treatment outcomes in pediatric Class III malocclusions. In a previous study, we achieved automatic classification of pediatric sagittal facial patterns by using lateral cephalometric radiographs and profile photographs, achieving accuracies of 94.05 and 85.49%, respectively [38]. A recent study of 37 patients suggested that the use of lateral cephalograms might help in prognosis prediction and treatment decisions for children with skeletal class Ⅲ malocclusions [41]. In order to achieve a more accurate and objective classification of pediatric Class III malocclusions, we collected a dataset of 4–14-year-old patients who met inclusion criteria. By using 46 representative cephalometric measurement features as the input parameters, we trained ten ML models and compared their performances. GPR produced the best results among these models, achieving an AUC value of 0.879. Therefore, we have chosen the GPR model for further analysis of the important features.

Specifically, ML models excelled in classifying dental Class III malocclusions, with KNN, OvR, Linear SVM, RBF SVM and GPR models achieving accuracies above 86.5% and specificities exceeding 91%. In contrast, skeletal malocclusion classification was intermediate, with accuracies ranging from 77 to over 88%. Functional malocclusion classification had the lowest performance with accuracies ranging from 80.5 to over 68% (Table S4). This discrepancy may be due to the more pronounced features of dental and skeletal malocclusions, whereas functional Class III malocclusion features have a tendency to locate between the skeletal and dental malocclusions, making them harder to differentiate.

From 46 cephalometric measurement features, we selected 14 significant factors which were likely to influence pediatric Class III malocclusion classification. These factors included age, NA-APo, SN-GoMe, Wits, Overjet, Go-Pog, SGn-FH, ANB, U1-NA, U1-Po, L1-Po, IMPA and AB-NPo encompassing the patients’ ages, vertical jaw relationships, growth patterns, upper and lower incisor angles as well as chin positions. The feature importance plot (Figure 4) highlights SN-GoMe, U1-NA, Overjet and ANB as the most influential features for classification. SN-GoMe has been identified as an important negative predictor for early functional Class III malocclusions and a significant predictor for adult orthognathic surgery demand [42], particularly in skeletal malocclusions characterized by steep mandibular planes. This aligns with our study, where the SN-GoMe was a crucial feature for distinguishing skeletal from functional Class III malocclusions. Regarding upper and lower incisor angles, the U1-NA angle played a significant role in distinguishing functional from skeletal malocclusions. Larger U1-NA angles can indicate more pronounced compensatory inclinations of the upper incisors, suggesting the initial presence of skeletal malocclusions. Overjet (coverage) also emerged as an important indicator for diagnosing dental malocclusions especially in cases where they primarily manifest as occurring at abnormal upper and lower incisor positions, slight lingual inclination of upper incisors as well as minimal overjets.

In sagittal dimensions, a Class III patient will typically exhibit a concave facial profile. The feature importance plot showed that the ANB angle, Wits and NA-APo angle are crucial variables for sagittal relationships in Class III malocclusions. The ANB angle has been reported to be one of the best predictors for relapse after Class III malocclusion treatment and a top predictor for facial type classification in children and adults [40,43,44]. In these cases, Wits assessment may work in conjunction with the ANB angle, as they belong to different reference systems and need simultaneous consideration. Some researchers have emphasized the NA-APo angle as an important predictor for diagnosing a dental Class III malocclusion, rather than the ANB angle [45,46]. Patients with larger NA-APo angles have higher risks of skeletal malocclusions (Figure 5c This suggests that the chin may reflect the presence of early abnormalities.

Our study has certain limitations. Firstly, the patient sample was restricted to a single center, which may limit the external validity and generalizability of our findings across different populations. Therefore, future research involving large-scale, multicenter, prospective studies is crucial for validating our findings. Secondly, this study utilized only cephalometric measurement data as input parameters, while clinical patient information typically includes other data such as facial photo and intraoral photographs as well as case histories. Subsequent studies could consider integrating the clinical data to the image information in order to enhance the robustness of the analysis performed. Thirdly, our study exclusively employed ML algorithms. Future investigations might explore methods such as radiomics and deep learning to automatically extract image features and combine them with cephalometric measurements by using deep neural networks for multimodal data fusion to further improve classification accuracy. Finally, future research could explore the potential application of AI-ML predictive models as clinical decision support systems for early treatment planning for Class III malocclusions. This could guide early intervention decisions, promote favorable jaw growth and reduce the risks and treatment difficulties associated with surgeries performed when the patients become older.

In summary, our study has two significant research implications. Firstly, it represents the first attempt to apply ML methods to the classification diagnosis of pediatric Class III malocclusions. Secondly, from an orthodontic perspective, we analyzed the importance of included features and validated clinically relevant characteristics from a ML standpoint. This will provide orthodontists with a valuable assessment reference for diagnosing pediatric Class III malocclusions, enabling correct early intervention measures, improving unfavorable jaw relationships, guiding harmonious development of the upper and lower jaws and promoting normal oral and facial development of young children.

## 5. Conclusions

ML-based methods can successfully achieve the classification diagnosis for pediatric Class III malocclusions. Among the models tested, the Gaussian Process Regression (GPR) model demonstrated the best classification performance, with an AUC value of 0.879. Specifically, the highest accuracy was achieved when classifying the dental Class III type, reaching 87.50%, followed by skeletal Class III, and the lowest classification ability was observed for functional Class III. Key indicators for diagnosing dental, functional and skeletal Class III malocclusions included the SN-GoMe, U1-NA, Overjet and ANB. This study suggests that combining ML with quantitative imaging analysis could enhance personalized diagnostic classification for pediatric Class III malocclusions and improve our understanding of the underlying growth mechanisms in the craniofacial region.

The strength of this study lies in its accurate classification of pediatric Class III malocclusions using ML models based solely on cephalometric measurement data. However, there are still some limitations. Firstly, the small sample size is a critical issue. Secondly, the information obtained solely from cephalometric data may be insufficient. Therefore, future work should involve constructing larger-scale, multicenter and prospective datasets in order to validate our findings. Additionally, exploring deep neural networks for learning from multimodal data could lead to better classification outcomes. While artificial intelligence (AI) aids in the classification diagnosis of Class III malocclusions, it lacks the reasoning process, potentially diverting the attention of clinicians from crucial details. Thus, AI-based diagnosis should serve as a potential auxiliary tool for clinicians rather than being used as a standalone technique.

## Figures and Tables

**Table 1:** Definitions of the 46 cephalometric features used in this study.

No.	Cephalometric Variables	Definition
1	SNA	The angle SN to point A (degrees)
2	SNB	The angle SN to point B (degrees)
3	ANB	The difference between angles SNA and SNB (degrees)
4	MP-SN	Angulation between the mandibular plane and the SN plane (degrees)
5	FH-MP	Angulation between the mandibular plane and the Frankfort plane (degrees)
6	SGn-FH	Sella gnathion to the Frankfort horizontal plane (degrees)
7	PP-GoGn	The angle between the PP and GoGn line (degrees)
8	OP-SN	Angulation between the functional occlusal plane and the SN plane (degrees)
9	PP-FH	Angulation between the pp plane and the Frankfort horizontal plane (degrees)
10	AB-NPo	The angle between the AB and N-Pog line (degrees)
11	NA-APo	The angle between the N-A and A-Pog line (degrees)
12	FH-NPo	The angle between the N-pog line and Frankfort horizontal plane (degrees)
13	S-N	Distance between S and N (mm)
14	Co-Po	The distance between two lines perpendicular to mandibular plane from Co and Pog (mm)
15	S-Go	Distance between S and Go (mm)
16	Go-Pog	The distance between two lines perpendicular to mandibular plane from Go and Pog (mm)
17	Go-Co	Distance between Go and Co (mm)
18	SVert-Co	The perpendicular distance from the Co to the line perpendicular to Frankfort horizontal plane through the S (mm)
19	Ptm-A	The distance between two lines perpendicular to Frankfort horizontal plane from Ptm and A (mm)
20	S-Ptm	The distance between two lines perpendicular to Frankfort horizontal plane from S and Ptm (mm)
21	Wits	The distance between two lines perpendicular to occlusal plane from A and B (mm)
22	ANSMe-NMe	The ratio of the length of ANSMe to that of NMe (%)
23	PFH-AFH	The ratio of the posterior face height to the anterior face height (%)
24	SGo-NMe	The ratio of the length of SGo to that of NMe (%)
25	SN-GoMe	The ratio of the length of the anterior skull base SN to that of the mandibular base GoMe (%)
26	IMPA	Mandibular incisor angulation to mandibular plane (degrees)
27	FMIA	Mandibular incisor angulation to Frankfort horizontal plane (degrees)
28	Overjet	The distance between two lines perpendicular to the functional occlusal plane from U1 and L1 (mm)
29	Overbite	The distance between two lines parallelled to the functional occlusal plane from U1 and L1 (mm)
30	U1-L1	The angle between the line through the long axis of the upper and lower central incisors (degrees)
31	U1-SN	Maxillary incisor angulation to SN plane (degrees)
32	U1-NA (mm)	The perpendicular distance from U1 to the NA line (mm)
33	U1-NA	Maxillary incisor angulation to NA plane (degrees)
34	L1-NB (mm)	The perpendicular distance from L1 to the NB line (mm)
35	L1-NB	Mandibular incisor angulation to NB plane (degrees)
36	U1-Apo (mm)	The perpendicular distance from U1 to the Apo line (mm)
37	L1-Apo (mm)	The perpendicular distance from L1 to the Apo line (mm)
38	Ptm-U6	The distance between two lines perpendicular to Frankfort horizontal plane from Ptm and U6 (mm)
39	FH-N′pog′	Angulation between the N′Pog′ and the Frankfort plane (degrees)
40	N Vert-Pog	The perpendicular distance from Pog′ to the line perpendicular to Frankfort horizontal plane through the N′ (mm)
41	N-Sn-Pog	The angle between the N′-Sn and Sn-Pog′ line (degrees)
42	UL-EP	The perpendicular distance from UL to the E-line (mm)
43	LL-EP	The perpendicular distance from LL the E-line (mm)
44	Z-Angle	The angle between the line of Pog′ to the most protuberant lip (upper or lower lip) and the Frankfort horizontal plane (degrees)
45	Sn to G Vert	The perpendicular ditance from Sn to the line perpendicular to Frankfort horizontal plane through the G (mm)
46	Si-H	The perpendicular distance from Si to the H line (mm)

**Table 2:** The class III malocclusion classification diagnostic reference criteria used in this study.

		Dental	Functional	Skeletal
**History**	Family history	None	None	Present
	Age at consultation	Early childhood	Early childhood	Later age
**Clinical Examination**	Occlusal relationship	Neutrocclusion	Neutrocclusion or beginning mesiocclusion	Mesiocclusion or complete mesiocclusion
	Overbite/Overjet (OB/OJ)	Shallow OJ, shallow OB	Deep OJ	Open bite or overbite tendency
	Mandibular retrusion	Possible	Possible	Not possible
	Posterior crossbite	Absent	Absent	Often present
	Dental crowding	Absent	Mild mandibular crowding	Severe maxillary crowding
	Mandibular deviation	Absent	Absent	Often present
**Cephalometric Analysis**	Incisor lip inclination	Maxillary incisors lingually inclined	Mandibular incisors labially inclined; Maxillary incisors normal or slightly labially inclined	Maxillary incisors labially inclined
	Jaw length	Normal	Normal or maxillary deficiency	Mandibular excess length
	Chin angle	Normal	Normal or increased	Increased
ANB angel	Normal	Normal or <0	<0	

**Table 3:** Machine learning model performance in the testing set.

	Model	Accuracy (%)	Specificity (%)	Precision (%)	Recall (%)	F1-Score (%)
1	Nearest Neighbors	82.33	84.76	65.52	65.12	65.17
2	L2 logistic (OvR)	83.00	83.40	68.85	63.09	64.92
3	Linear SVM	82.00	83.77	65.38	63.27	64.09
4	RBF SVM	82.33	84.57	66.01	64.14	63.95
5	Gaussian Process	81.33	83.15	63.13	61.09	61.76
6	Decision Tree	78.33	82.44	56.48	57.95	57.04
7	Neural Net	79.33	84.06	61.82	62.26	61.77
8	Random Forest	81.00	84.12	61.98	60.92	61.39
9	QDA	81.33	84.92	65.17	66.01	65.54
10	xgboost	79.67	85.64	63.16	60.42	61.38

## Data Availability

We declare that data cannot be obtained due to privacy or ethical restrictions. All the data generated and analyzed during this study are included in this published article (and its Supplementary Information files).

## Supplementary Materials

The following supporting information can be downloaded at: https://www.mdpi.com/article/10.3390/children11070762/s1, Figure S1: Cephalometric Landmarks used in this study; Figure S2: The confusion matrix plots for 10 ML models; Table S1: Demographic information Mean ± SD and n (%); Table S2: Cephalometric variables and age for the participants Mean ± SD (°) (mm) (%); Table S3: Selected features; Table S4: Accuracy, Sensitivity, Specificity, Recall and F1-score of ten CNNs with dental, fuctional and skeletal Classification diagnosis.
