# Supplementary material for: Machine Learning Algorithms for the Diagnosis of Class III Malocclusions in Children
Source: Children (Basel). 2024 Jun 24;11(7):762. doi: 10.3390/children11070762 (PMC11274672; doi:10.3390/children11070762)
Supplement: Supplementary file 1 [file children-11-00762-s001.zip › children-3001828-supplementary.pdf]

Machine Learning Algorithms for Prediction of the classification diagnosis in Children with Class III Malocclusions

Bohui Liang [a#], Ling Zhao [b#], Xiaozhi Chen [c], Juneng Huang [a], Shuixue Mo [b], Min Gu [d], Na Kang [b], Shaohua Song [b], Xuejun Zhang [a], and Min Tang [be*]

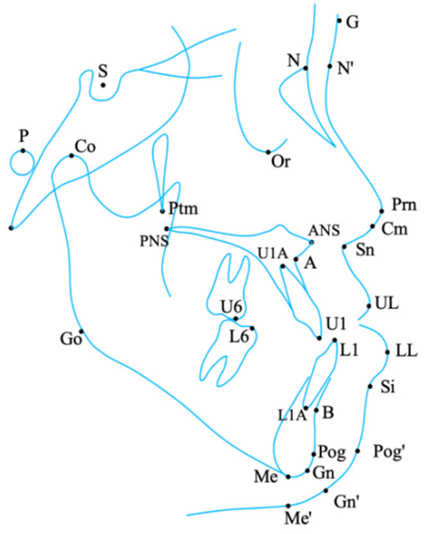

Figure S1.

Cephalometric Landmarks used in this study.

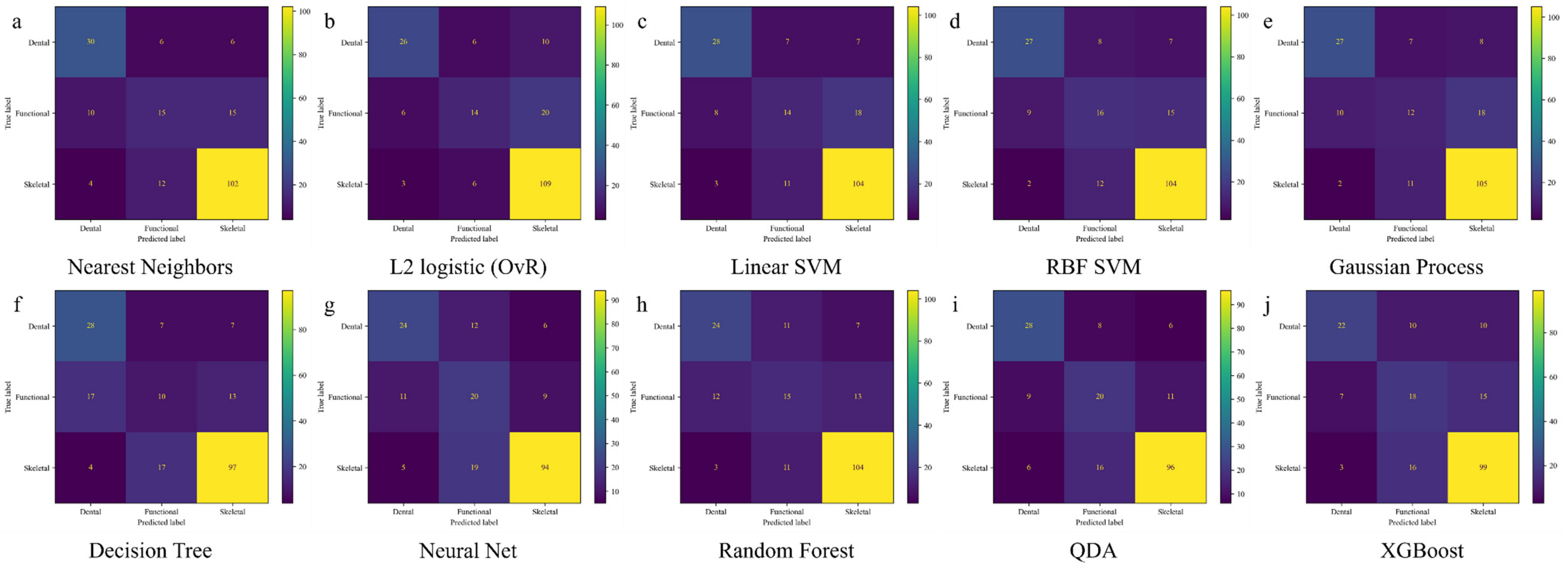

Figure S2.

The confusion matrix plots for 10 ML models

Table S1. Demographic information Mean±SD and n (%)

Parameters	Dental	Functional	Skeletal	Total
**Numbers**	140 (21.02)	132 (19.82)	394 (59.16)	666
**Male**	76 (11.41)	71 (10.66)	210 (31.53)	357 (53.60)
**Female**	64 (9.61)	61 (9.16)	184 (27.63)	309 (46.40)
**Age (y, mean ± SD)**	9.1±2.0	10.3±2.2	11.4±1.6	10.68±2.04

Table S2. Cephalometric variables and age for the participants Mean±SD (°) (mm) (%)

No.	Cephalometric variables	Mean±SD			p			
		Dental (n=140)	Functional (n=132)	Skeletal (n=394)	Dental vs. Functional	Dental vs. Skeletal	Functional vs.Skeletal	Anova
1	age	9.1±2.0	10.3±2.2	11.4±1.6	<0.001	<0.001	<0.001	0.002
2	SNA	81.5±3.3	80.2±3.4	80.7±3.3	<0.001	0.016	0.08	0.003
3	SNB	80.1±3.5	80.0±3.4	83.5±3.7	0.816	<0.001	<0.001	<0.001
4	ANB	1.5±1.9	0.2±1.8	-2.8±2.3	<0.001	<0.001	<0.001	<0.001
5	MP-SN	35.0±5.3	35.8±5.0	33.3±5.2	0.206	<0.001	<0.001	<0.001
6	FH-MP	26.8±4.4	27.0±4.9	25.5±5.0	0.8	0.008	0.004	0.002
7	SGn-FH	60.9±2.6	59.4±3.3	58.3±3.3	<0.001	<0.001	<0.001	<0.001
8	PP-GoGn	24.0±4.3	24.2±4.2	22.6±4.6	0.728	<0.001	<0.001	<0.001
9	OP-SN	18.3±4.7	19.0±4.5	15.4±4.7	0.175	<0.001	<0.001	<0.001
10	PP-FH	1.2±2.8	1.0±3.0	1.4±3.1	0.578	0.458	0.163	0.348

11	AB-NPo	-1.5±2.6	0.4±2.4	4.3±3.2	**<0.001**	**<0.001**	**<0.001**	**<0.001**
12	NA-APo	4.0±4.3	1.4±4.5	-5.5±5.5	**<0.001**	**<0.001**	**<0.001**	**<0.001**
13	FH-NPo	87.8±2.7	88.3±3.1	91.1±3.4	0.204	**<0.001**	**<0.001**	**<0.001**
14	S-N	58.9±2.9	58.4±3.6	59.6±3.6	0.218	**0.046**	**<0.001**	**0.001**
15	Co-Po	90.0±6.6	87.8±7.5	95.4±7.8	**0.019**	**<0.001**	**<0.001**	**<0.001**
16	S-Go	66.4±5.4	62.9±6.4	67.4±6.1	**<0.001**	0.076	**<0.001**	**<0.001**
17	Go-Pog	64.6±4.6	62.2±4.9	68.3±5.7	**<0.001**	**<0.001**	**<0.001**	**<0.001**
18	Go-Co	49.0±4.5	46.5±5.1	50.0±4.6	**<0.001**	**0.039**	**<0.001**	**<0.001**
19	S Vert-Co	9.8±2.1	8.8±2.3	9.9±2.3	**<0.001**	0.484	**<0.001**	**<0.001**
20	Ptm-A	39.8±2.6	38.3±3.0	39.5±3.1	**<0.001**	0.242	**<0.001**	**<0.001**
21	S-Ptm	18.2±1.9	18.4±2.2	18.1±2.2	0.368	0.777	0.173	0.394
22	Wits	-4.1±2.7	-5.4±2.3	-8.2±3.2	**<0.001**	**<0.001**	**<0.001**	**<0.001**
23	ANSMe-NMe (%)	54.7±1.8	54.5±2.0	54.4±1.9	0.334	0.197	0.923	0.423
24	PFH-AFH (%)	69.0±7.0	68.4±7.3	71.3±7.1	0.487	**<0.001**	**<0.001**	**<0.001**
25	SGo-NMe (%)	64.4±4.0	63.4±3.4	64.6±3.8	**0.03**	0.527	**0.001**	**0.005**
26	SN-GoMe (%)	97.2±6.6	100.1±6.9	92.4±6.1	**<0.001**	**<0.001**	**<0.001**	**<0.001**
27	IMPA	91.9±7.3	88.1±5.7	86.4±6.7	**<0.001**	**<0.001**	**0.009**	**<0.001**

28	FMIA	61.2±6.5	64.9±5.9	68.1±6.9	**<0.001**	**<0.001**	**<0.001**	**<0.001**
29	Overjet	0.0±2.1	-1.8±1.4	-2.5±1.7	**<0.001**	**<0.001**	**<0.001**	**<0.001**
30	Overbite	0.9±1.4	2.0±2.0	2.8±2.3	**<0.001**	**<0.001**	**<0.001**	**<0.001**
31	U1-L1	127.5±11.2	136.4±11.6	131.3±9.8	**<0.001**	**<0.001**	**<0.001**	**<0.001**
32	U1-SN	105.5±8.3	99.6±9.6	109.1±7.0	**<0.001**	**<0.001**	**<0.001**	**<0.001**
33	U1-NA(mm)	3.6±2.6	2.0±2.4	5.0±2.3	**<0.001**	**<0.001**	**<0.001**	**<0.001**
34	U1-NA	24.0±7.7	19.6±8.6	28.3±6.2	**<0.001**	**<0.001**	**<0.001**	**<0.001**
35	L1-NB(mm)	5.5±2.2	4.3±2.1	4.4±2.1	**<0.001**	**<0.001**	0.94	**<0.001**
36	L1-NB	27.1±6.0	23.9±5.2	23.2±5.8	**<0.001**	**<0.001**	0.215	**<0.001**
37	U1-Apo(mm)	4.9±2.5	2.5±2.2	3.1±2.3	**<0.001**	**<0.001**	**0.007**	**<0.001**
38	L1-Apo(mm)	5.0±2.2	4.6±1.9	6.2±2.1	0.115	**<0.001**	**<0.001**	**<0.001**
39	Ptm-U6	15.1±3.7	14.3±3.6	15.9±3.8	0.096	**0.029**	**<0.001**	**<0.001**
40	FH-N'pog'	91.8±3.1	92.5±3.5	95.6±3.8	0.112	**<0.001**	**<0.001**	**<0.001**
41	N Vert-Pog	2.9±5.0	3.8±5.3	9.0±6.2	0.194	**<0.001**	**<0.001**	**<0.001**
42	N-Sn-Pog	14.4±4.5	11.8±4.6	7.1±5.7	**<0.001**	**<0.001**	**<0.001**	**<0.001**
43	UL-EP	1.2±1.8	0.3±1.8	-0.9±2.1	**<0.001**	**<0.001**	**<0.001**	**<0.001**
44	LL-EP	3.8±2.1	3.4±2.2	3.3±2.4	0.142	**0.04**	0.814	0.117

45	Z-Angle	71.4±6.2	71.8±6.5	74.0±7.8	0.59	**<0.001**	**0.003**	**<0.001**
46	Sn to G Vert	3.3±3.5	2.2±3.3	3.0±3.7	**0.01**	0.395	**0.023**	**0.025**
47	Si-H	1.6±1.4	1.5±1.2	1.1±1.5	0.378	**<0.001**	**0.007**	**<0.001**

Table S3. Selected features.

Index	Feature	Index	Feature	Index	Feature
1	age	6	Go-Pog	11	L1-APo(mm)
2	NA-APo	7	SGn-FH	12	IMPA
3	SN-GoMe	8	ANB	13	Si-H
4	Wits	9	U1-NA	14	AB-NPo
5	Overjet	10	U1-APo(mm)		

Table S4. Accuracy, Sensitivity, Specificity, Recall and F1 -score of ten CNNs with dental,fuctional and skeletal Classification diagnosis .

Model		Accuracy (%)	Specificity (%)	Precision(%)	Recall	F1 -score
Nearest Neighbors	Dental	87.00	91.14	68.18	71.43	69.77
	Functional	78.50	88.75	45.45	37.50	41.10
	Skeletal	81.50	74.39	82.93	86.44	84.65
L2 logistic (OvR)	Dental	87.50	94.30	74.29	61.90	67.53
	Functional	81.00	92.50	53.85	35.00	42.42
	Skeletal	80.50	63.41	78.42	92.37	84.82
Linear SVM	Dental	87.50	93.04	71.79	66.67	69.17
	Functional	78.00	88.75	43.75	35.00	38.89
	Skeletal	80.50	69.51	80.62	88.14	84.21
RBF SVM	Dental	87.00	93.04	71.05	64.29	67.50
	Functional	78.00	87.50	44.44	40.00	42.11
	Skeletal	82.00	73.17	82.54	88.14	82.25
Gaussian Process_test	Dental	86.50	92.41	69.23	64.29	66.67
	Functional	77.00	88.75	40.00	30.00	34.29
	Skeletal	80.50	68.29	80.15	88.98	84.34

Decision Tree	Dental	82.50	86.71	57.14	66.67	61.54
	Functional	73.00	85.00	29.41	25.00	27.03
	Skeletal	79.50	75.61	82.91	82.20	82.55
Neural Net	Dental	83.00	89.87	60.00	57.14	58.54
	Functional	74.50	80.62	39.22	50.00	43.96
	Skeletal	80.50	81.71	86.24	79.66	82.82
Random Forest	Dental	83.50	90.51	61.54	57.14	59.26
	Functional	76.50	86.25	40.54	37.50	38.96
	Skeletal	83.00	75.61	83.87	88.14	85.95
QDA_	dental	85.50	90.51	65.12	66.67	65.88
	functional	78.00	85.00	45.45	50.00	47.62
	skeletal	80.50	79.27	84.96	81.36	83.12
xgboost	dental	85.00	93.67	68.75	52.38	59.46
	functional	76.00	93.75	40.91	45.00	42.86
	skeletal	78.00	69.51	79.84	83.90	81.82
